# Genetic disruption of the pH_i_-regulating proteins Na^+^/H^+^ exchanger 1 (SLC9A1) and carbonic anhydrase 9 severely reduces growth of colon cancer cells

**DOI:** 10.18632/oncotarget.14379

**Published:** 2016-12-30

**Authors:** Scott K. Parks, Yann Cormerais, Jerome Durivault, Jacques Pouyssegur

**Affiliations:** ^1^ Medical Biology Department, Centre Scientifique de Monaco (CSM), Monaco; ^2^ Institute for Research on Cancer & Aging (IRCAN), CNRS, INSERM, Centre A. Lacassagne, University of Nice-Sophia Antipolis, Nice, France

**Keywords:** Na^+^/H^+^-exchanger (NHE1), Carbonic Anhydrase 9 (CA9), tumor hypoxia, pH regulation, tumor growth

## Abstract

Hypoxia and extracellular acidosis are pathophysiological hallmarks of aggressive solid tumors. Regulation of intracellular pH (pH_i_) is essential for the maintenance of tumor cell metabolism and proliferation in this microenvironment and key proteins involved in pH_i_ regulation are of interest for therapeutic development. Carbonic anhydrase 9 (CA9) is one of the most robustly regulated proteins by the hypoxia inducible factor (HIF) and contributes to pH_i_ regulation. Here, we have investigated for the first time, the role of CA9 via complete genomic knockout (ko) and compared its impact on tumor cell physiology with the essential pH_i_ regulator Na^+^/H^+^ exchanger 1 (NHE1). Initially, we established NHE1-ko LS174 cells with inducible CA9 knockdown. While increased sensitivity to acidosis for cell survival in 2-dimensions was not observed, clonogenic proliferation and 3-dimensional spheroid growth in particular were greatly reduced. To avoid potential confounding variables with use of tetracycline-inducible CA9 knockdown, we established CA9-ko and NHE1/CA9-dko cells. NHE1-ko abolished recovery from NH_4_Cl pre-pulse cellular acid loading while both NHE1 and CA9 knockout reduced resting pH_i_. NHE1-ko significantly reduced tumor cell proliferation both in normoxia and hypoxia while CA9-ko dramatically reduced growth in hypoxic conditions. Tumor xenografts revealed substantial reductions in tumor growth for both NHE1-ko and CA9-ko. A notable induction of CA12 occurred in NHE1/CA9-dko tumors indicating a potential means to compensate for loss of pH regulating proteins to maintain growth. Overall, these genomic knockout results strengthen the pursuit of targeting tumor cell pH regulation as an effective anti-cancer strategy.

## INTRODUCTION

Solid tumors are distinguished from normal tissues by the presence of an acidic and hypoxic extracellular environment arising from exacerbated and predominantly glycolytic tumor cell metabolism. Thus, both hypoxia-regulated and metabolism-related proteins are being pursued as cancer specific targets for therapeutic development (For a recent review refer to [1]). A key advantage that tumor cells posses in this harsh environment is an extremely efficient intracellular pH (pH_i_) regulating system. Maintenance of pH_i_ is essential for a wide range of cellular functions (for a recent review of a range of pathologies impacted by pH disturbances see [2]) and tumor cells have proven to be exceptional at regulating their pH_i_ in the face of extracellular (pH_e_) acidosis. Consequently, disruption of pH_i_ regulation in tumor cells has been of great interest in the past decade [3].

After cellular buffering systems are exhausted (for a review of the importance of cellular buffering see [4]) secretion of H^+^ or uptake of HCO_3_^-^ is required to prevent acidification of pH_i_. The most robust and well described H^+^ secreting protein found in all cells is the Na^+^/H^+^-exchanger 1 (NHE1/SLC9A1). Early enthusiasm for the targeting of tumor cell pH_i_ regulation as an anti-cancer strategy was generated from mutated cells exhibiting exclusively glycolysis or respiration in the presence or absence of NHE1. Both *in vitro* and tumor xenografts using these cells demonstrated the essential nature of pH_i_ regulation via NHE1 for both tumor initiation and growth [5–9]. This led to translational oncology studies using pharmacological inhibitors of NHE1 [10–12]. Unfortunately, toxicity due to NHE1 inhibitors in concomitant cardiac clinical trials resulted in their abandonment in all areas of the clinic (see [3, 13] for a more extensive discussion). Despite this, NHE1 continues to be investigated for its importance in tumor cell progression and in particular cell migration/metastasis and blockade of the H^+^ secreting strategy in cancer cells remains an attractive therapeutic target [14–17].

Contributions of CO_2_/HCO_3_^-^ balance to tumor pH_i_ and pH_e_ surged to the forefront of the literature following the discovery that the extracellular facing carbonic anhydrase 9 (CA9) is robustly regulated by hypoxia [18]. CA9 expression in normal physiology is limited to a small region of the gastrointestinal tract whereas it is overexpressed in numerous solid tumors and acts as a poor prognostic factor (for an extensive list see [19]). Confirmation that CA9 contributes to the control of pH_i_ regulation in addition to acidification of pH_e_ [20–23] prompted a widespread effort to develop pharmacological agents to target this almost exclusive cancer protein. Recent support for importance of HCO_3_^-^ uptake in tumor cells has strengthened the need to further understand CA9 activity in the tumor microenvironment [24, 25]. The majority of pre-clinical data for CA9 has involved mixed use of shRNA and various inhibitors with the greatest success being realized in syngeneic mouse tumor models [26]. Despite the intense interest in small molecule inhibitor development targeting CA9 (for extensive review refer to [19, 27]) no cellular knockout models have been reported to serve as validation tools in drug development. Progress has been made however and clinical trials targeting CA9 in solid tumors are currently ongoing [27].

Our goal in this study was two-fold. An unresolved question stemming from earlier work in our lab involving CA9 knockdown was whether NHE1 inhibition would synergize with disruption of CO_2_/HCO_3_^-^ regulating systems. Limitations of the ability to use NHE1 specific inhibitors and tetracycline for induction of shRNA [28, 29] in mouse models led us to develop complete allelic disruption of either NHE1 (NHE1-ko), CA9 (CA9-ko) or both (NHE1/CA9-dko). This gene disruption approach validates the importance of CA9 in both *in vitro* and *in vivo* tumor progression, particularly in hypoxia. Interestingly, we observed that NHE1-ko has a dramatic impact on tumor cell growth both in normoxia and hypoxia however there is not a clear synergy with combined NHE1/CA9-dko potentially due to a strong concomitant induction of CA12.

## RESULTS

### NHE1 knockout development

**Figure 1:**
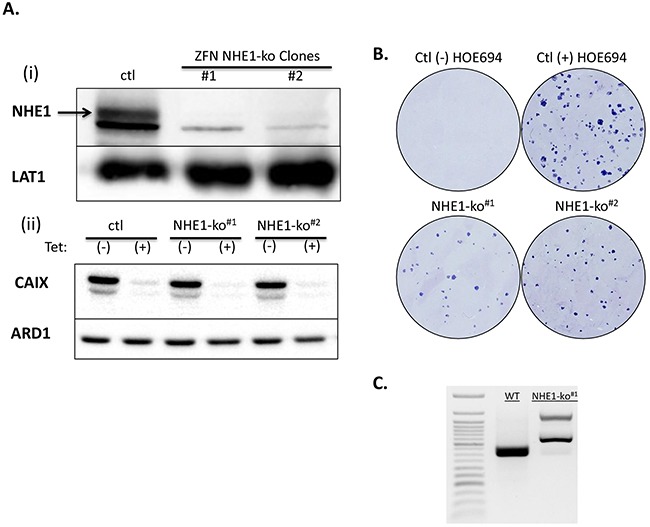
Generation of NHE1 knockouts (NHE1-ko) in LS174 cells via genomic disruption using ZFN targeting **Ai.** WB analysis of NHE1-ko generation in clones derived from ZFN transfection and subsequent H+-suicide. LAT1 was used as an internal loading control as WB was performed on membrane enriched cell extracts. **Aii.** WB confirming maintenance of the tetracycline (tet) inducible knockdown of CA9 (CA9-kd) in NHE1-ko cells. **B.** H+-suicide using LiCl confirmed functional removal of NHE1 activity in NHE1-ko#1&#2 cells compared to WT cells as determined by clonogenicity survival assays. The NHE1 specific inhibitor HOE694 (100μM) was applied to control cells for comparison purposes. **C.** Genomic PCR screening was used to confirm the mutated alleles introduced via ZFN. Genomic sequence data is provided in Supplementary Data (Supplementary Figure 1).

NHE1 knockout (NHE1-ko) mutations were achieved in LS174pTerCA9 [20] cells using Zinc Finger Nucleases (ZFN). Western blot analysis revealed that the glycosylated band of ∼115kDa is the specific band for NHE1 with a non-specific band at 100kDa (Figure 1A). Cellular membrane enrichment protocols were performed to improve NHE1 signal with another membrane protein (LAT1) serving as an internal loading control. NHE1-ko clones (named NHE1-ko^#1&#2^) maintained the tetracycline (tet) inducible shRNA knockdown (kd) of CA9 (Figure 1A lower panel). The LiCl H^+^-suicide technique, which takes advantage of the reversibility of Li^+^ transport via NHE1 to acid load cells [7] was used to confirm functional knockout of NHE1 activity in clonogenicity survival assays for NHE1-ko^#1&#2^ cells (Figure 1B). The NHE1 specific inhibitor HOE694 (100μM) was used as a control during LiCl H^+^-suicide experiments (Figure 1B). Therefore, although LS174 cells express mRNA for NHE2 and NHE3 (Supplementary Figure 2), NHE1 appears to carry the dominant NHE activity in these cells. Effects of NHE1-ko on pH_i_ regulation are discussed below. Genomic PCR screening and sequencing in the ZFN targeting region for NHE1 was performed to confirm disruption in two alleles (Figure 1C and Supplementary Figure 1).

**Figure 2:**
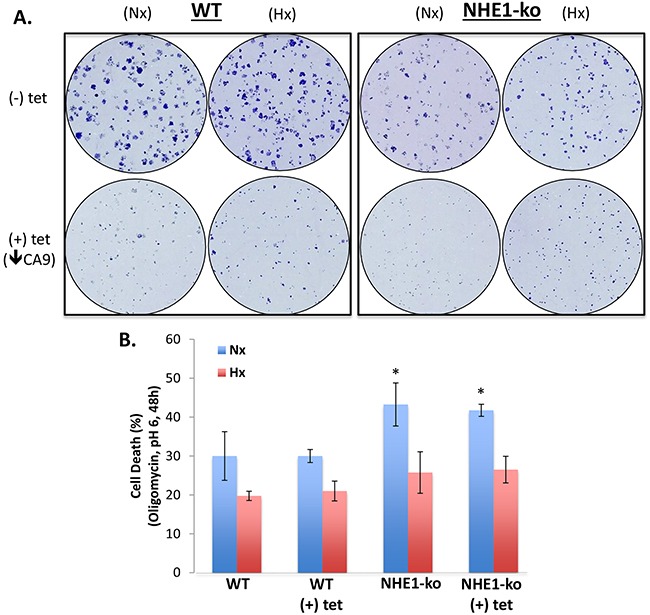
NHE1-ko and CA9-kd reduces clonogenic proliferation but does not substantially affect cell survival during acidic stress **A.** Clonogenicity in Nx and Hx conditions revealed a decreased proliferation rate in NHE1-ko cells compared to WT. CA9-kd reduced clonogenic proliferation more dramatically than NHE1-ko in both Nx and Hx. **B.** NHE1-ko marginally increased cell death upon exposure to extracellular acidosis and the OXPHOS inhibitor oligomycin (1μg/ml) in Nx only while CA9-kd did not affect cell survival in isolation or in combination with NHE1-ko (Nx and Hx). Significant differences compared to Ctl (-) tet are represented by an ‘*’ for Nx conditions (p<0.05). No significant differences were observed between Hx conditions.

Clonogenicity assays performed in normoxia (Nx) and hypoxia (Hx) revealed a reduction in proliferation with NHE1-ko but not to the same extent as tet-inducible CA9-kd (Figure 2A). A more extensive analysis of cell proliferation is provided below. Survival in acidic conditions was then assessed to determine if loss of NHE1 function renders LS174 cells more susceptible to acidic stress. Previously, we had demonstrated the survival advantage provided by Hx during acidosis in 2-dimensional culture conditions [30]. We pursued a similar protocol for NHE1-ko cells and observed that there was minimal impact on cell survival at a wide range of acidic pH exposures and time points (data not shown). As LS174 cells have proven to be extremely resistant to acidosis [30], we exposed them to a drastic acidic treatment in combination with oligomycin (Figure 2B). CA9-kd did not have an impact on cell survival in either Nx or Hx. NHE1-ko increased cell death slightly (10%) in Nx conditions with no noticeable effect in Hx (Figure 2B). No synergy was observed with combined NHE1-ko and CA9-kd.

### Impact of NHE1-ko and CA9-kd on 3-dimensional growth

**Figure 3:**
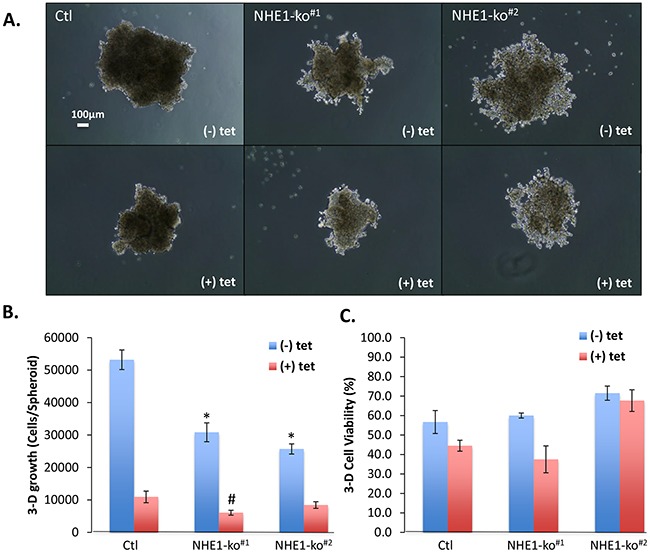
NHE1-ko and CA9-kd significantly impact 3-D spheroid growth characteristics **A.** 3-D spheroid growth (pHe 6.7) demonstrated reduced proliferation and altered morphology in NHE1-ko cells. CA9-kd (+tet) dramatically reduced 3-D proliferation with no apparent morphological alterations. Two independent NHE1-ko clones are presented to indicate the consistency of NHE1-ko. **B.** Summary of combined experiments showing significant reductions in 3-D proliferation with NHE1-ko and even further decreases with CA9-kd. A synergistic effect of NHE1-ko and CA9-kd was only observed in one NHE1-ko cell line. Statistical differences compared to Ctl (-tet) are represented by an ‘*’ while an ‘#’ represents differences compared to Ctl (+tet) (p<0.05) C. Summary of cell viability in 3-D growth assays showing minimal impacts on viability with either NHE1-ko or CA9-kd.

3-dimensional (3-D) spheroid growth experiments were performed to mimic aspects of the tumor microenvironment including hypoxia as has been previously established [20, 24]. NHE1-ko dramatically altered the ability of LS174 cells to form compact spheroids resulting in a more diffuse morphological phenotype (Figure 3A). Differential spheroid growth was observed in external pH of 7.4 vs. 6.7 with maintenance of phenotypic differences between WT and NHE1-ko cells (Supplementary Figure 3A). NHE1-ko alone reduced 3-D proliferation by ∼50% while CA9-kd reduced growth to a greater degree than NHE1-ko (Figure 3A, 3B). CA9-kd in spheroids was confirmed by WB analysis of protein extracts obtained at the time of cell counting (Supplementary Figure 3B). Despite the considerable impact on 3-D proliferation, appreciable differences in cell viability were not observed with either NHE1-ko or CA9-kd (Figure 3C).

### CA9 knockout development, cell proliferation and pH_i_ regulation

**Figure 4:**
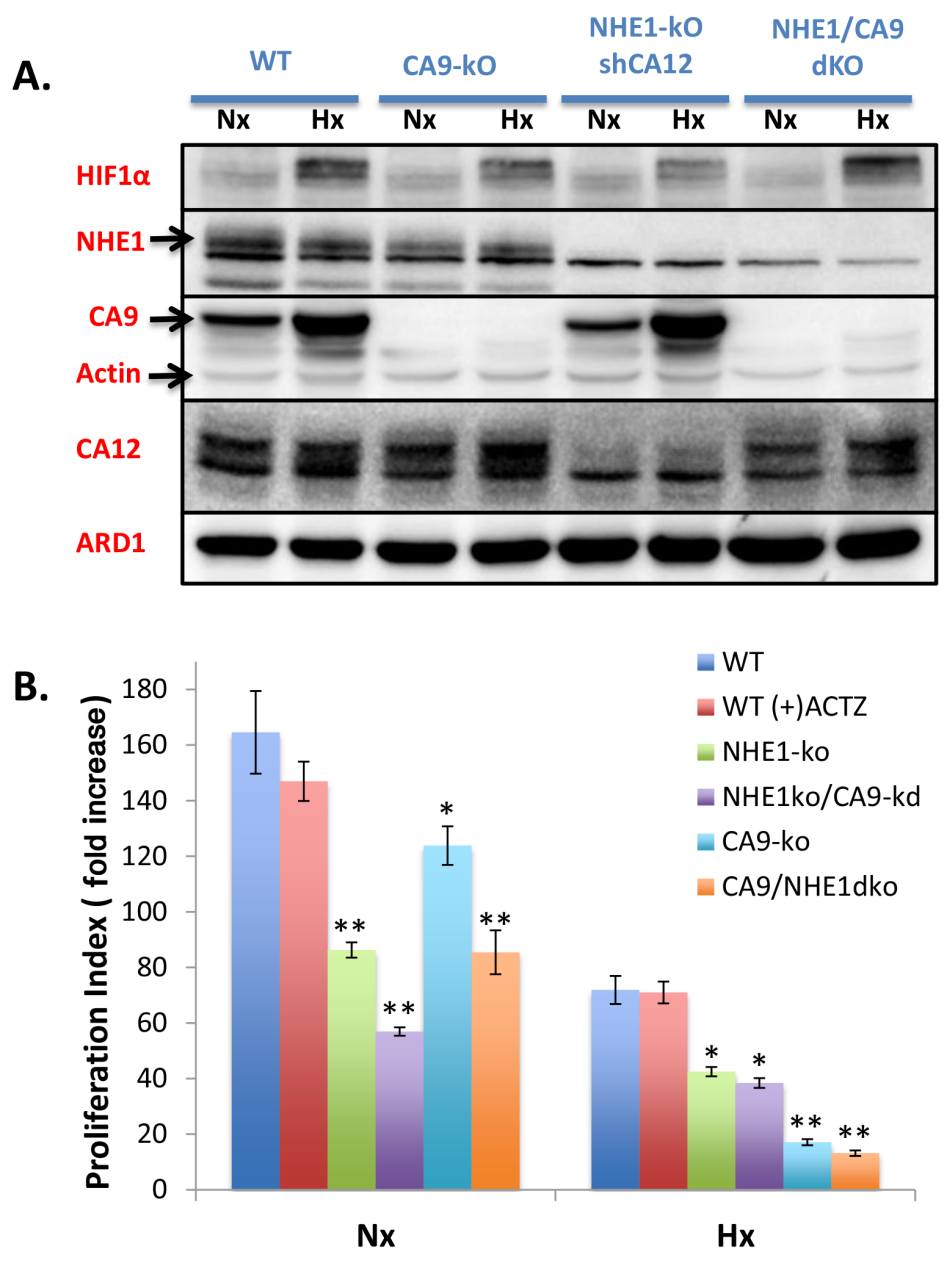
Generation of CA9-ko cells using CRISPR-cas9 to create single and NHE1/CA9 double knockout cell lines: impact on proliferation in Nx and Hx **A.** WB showing protein expression patterns for HIF1, NHE1, CA9, CA12 and loading controls (Actin, ARD1) in CA9-ko, NHE1-ko and NHE1/CA9-dko generated cells in comparison to WT cells from Nx and Hx growth conditions. **B.** Cellular proliferation assays from day 6 represented as fold increase standardized to the starting number of cells at 24h post-plating. Proliferation in Nx and Hx are presented on the same figure. The general CA inhibitor acetazolamide (ACTZ, 100μM) was used for comparative purposes. Statistical differences are indicated by an asterisk ‘*’ as compared to WT cells with analyses occurring in Nx and Hx assays individually. P values less than 0.05 are represented as ‘*’ while p values less than 0.001 are represented by ‘**’. Proliferation data were obtained from duplicate measurements on 3-4 independent experiments.

In light of recent concerns over the use of tetracyclines due to their inhibition of mitochondrial function [28, 29], we wished to develop CA9 knockout cells (CA9-ko) to avoid confounding metabolic disruptions when assessing the combined disruption of CA9 and NHE1. CA9-ko cells were created from WT cells using CRISPR-cas9 and subsequent ZFN targeting of NHE1 was performed to obtain NHE1/CA9 double knockout (dko) cells. Western blot analysis was used to observe the different protein knockouts achieved (Figure 4A). Genomic sequencing confirmed the knockout mutations introduced by CRISPR-Cas9 (Supplementary Figure 1). Cell proliferation assays performed in Nx and Hx revealed classical exponential growth curves with data from day 6 being represented for simplicity (Figure 4B). WT cells were unaffected by presence of the general CA inhibitor acetazolamide (ACTZ, 100μM) (Figure 4B). NHE1-ko significantly reduced cell proliferation by a similar magnitude in Nx and Hx compared to WT cells while CA9-ko dramatically reduced proliferation in Hx but to a much lesser extent in Nx. Combining CA9-kd (tetracycline) in NHE1-ko cells significantly decreased proliferation in Nx but did not alter growth in Hx. NHE1/CA9-dko cells grew at the same rate as single NHE1-ko cells in Nx while growing at a slightly lower but non-significant rate in Hx as compared to CA9-ko cells (Figure 4B).

**Figure 5:**
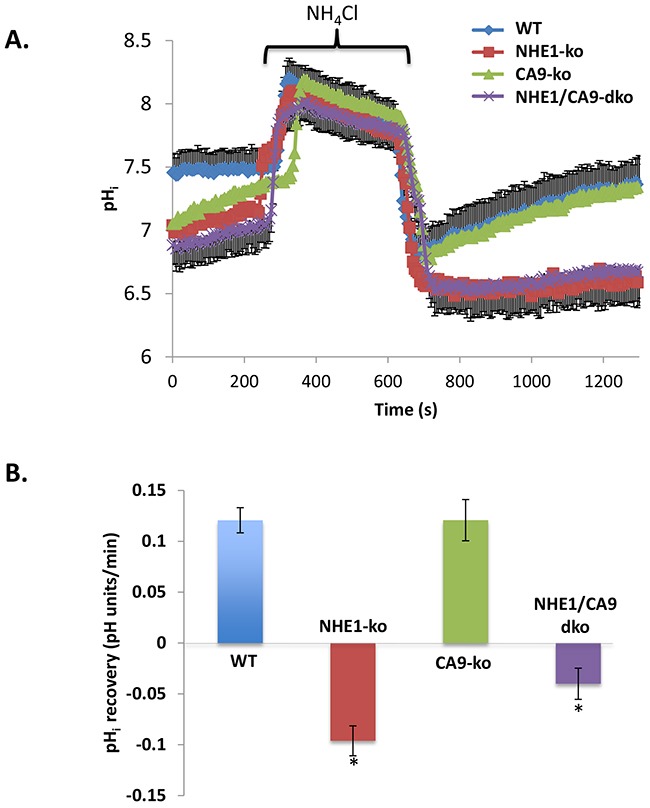
NHE1-ko abolishes HCO3- independent pHi recovery from an acid-load while altering resting pHi along with CA9-ko **A.** NH4Cl pre-pulse was used to acidify pHi and observe Na+-dependent pHi recovery in WT, NHE1-ko, CA9-ko and NHE1/CA9-dko cells. Each curve represents averaged values from a representative set of experiments. To improve ease of visualization, error bars are only provided for WT and NHE1/CA9-dko cells (-/+ SD). **B.** Statistical summary of pHi recovery rates in different cell types following acidosis. Recovery rates were calculated from the initiation of pHi recovery (see methods for further details). Values that are significantly different from WT cells are noted by an ‘*’. The total number of cells used for summary analyses were as follows: WT n=255, NHE1-ko n=178, CA9-ko n=91, NHE1/CA9-dko n=133.

Cells grown in hypoxia were monitored for resting pH_i_ and pH_i_ recovery characteristics following acid-loading with NH_4_Cl pre-pulse using the pH_i_ fluorescent indicator SNARF-1 and confocal microscopy. NHE1-ko, CA9-ko and NHE1/CA9-dko cells displayed a lower resting pH_i_ (between 0.2-0.4 pH units) compared to WT cells (Figure 5A). NHE1-ko completely abolished pH_i_ recovery from NH_4_Cl pre-pulse induced acidosis while expression of CA9 did not alter pH_i_ recovery (Figure 5A). Analysis of pH_i_ recovery rates indicated the essential nature of NHE1 for recovery from intracellular acidosis (Figure 5B) despite the gene expression of other NHE isoforms mentioned above (NHE2/3; Supplementary Figure 2). The NHE1 specific inhibitor HOE694 was used to confirm the absence of pH_i_ recovery from NH_4_Cl induced acidosis (data not shown).

### NHE1-ko and CA9-ko dramatically reduce growth of tumor xenografts

**Figure 6:**
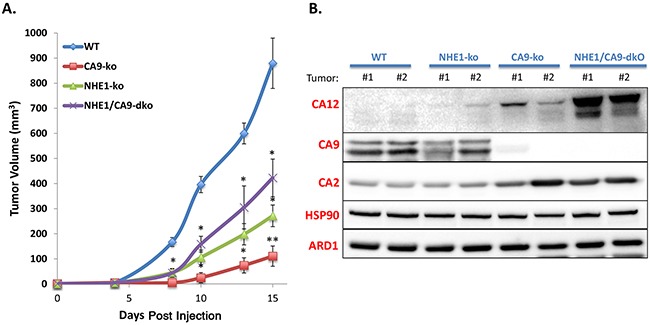
Knockout of NHE1 and CA9 significantly impairs tumor xenograft progression in a non-synergistic manner **A.** Tumor growth curves (mm3) over time for mice injected with WT, CA9-ko, NHE1-ko or NHE1/CA9-dko cells. Statistical differences (p<0.05) compared to WT tumors are represented by ‘*’. ‘**’ Represents statistical differences from both NHE1-ko and NHE1/CA9-dko tumors. **B.** WB analysis of tumor extracts for carbonic anhydrase isoforms 2, 9 and 12 (CA 2/9/12). Two protein-loading controls (ARD1 and HSP90) are provided for comparison purposes. 2 independent tumors are represented for each cell type. Additional tumor extracts were used in other WB to confirm these results.

As NHE1-ko cells demonstrated a notable disruption of 3-D spheroid growth, we performed tumor xenograft studies to observe the impact in an *in vivo* model. Tumor xenograft initiation was reduced by 40% in CA9-ko and NHE1/CA9-dko groups while 100% initiation occurred in WT and NHE1-ko tumors. NHE1-ko reduced tumor growth to an even greater extent (∼70%) than what was observed for *in vitro* cell growth (Figure 6A). CA9-ko resulted in the most substantial tumor growth reduction (∼90%) while NHE1/CA9-dko tumors grew at a surprisingly greater rate than both NHE1-ko and CA9-ko tumors. Protein extracts from tumor xenografts confirmed that the NHE1 and CA9 knockout phenotype induced by ZFN and CRISPR-cas9 was maintained (Figure 6B and Supplementary Figure 4). In addition, a striking increase of CA12 expression was observed in NHE1/CA9-dko tumor extracts (Figure 6B). CA2 was also increased in CA9-ko tumors and this increase was maintained in NHE1/CA9-dko tumors (Figure 6B).

## DISCUSSION

Tumor cell pH regulation has been a major theme in the understanding of cellular function within the tumor microenvironment. It is now clear that hypoxia drives numerous adaptations that protect tumor cells in acidic surroundings. These hypoxic alterations combine with inherent cellular machinery to provide a robust pH_i_ regulating capacity. Thus, the essential nature of stable pH_i_ for cellular function has led to the desire to disrupt pH_i_ regulation in cancer cells for novel therapeutic development. CA9 has been at the forefront of therapeutic development due to its nearly exclusive tumor expression pattern and induction by hypoxia.

Studies involving CA9 knockdown from our group and others established that CA9 contributes to tumor cell proliferation *in vitro* and *in vivo* and this has been linked to its role in regulating pH_i_ [20, 26, 31]. CA9 also plays a role in the metastatic capacity of tumor cells due to its catalytic generation of H^+^ in the extracellular space [26, 32, 33]. CA9 is also now implicated as an important protein in the generation of cancer stem cells (CSCs) [34–37]. Furthermore, CA9 disruption has been shown to be effective individually and to synergize with classical therapeutic approaches such as radiation [38, 39] and chemotherapy [31]. Our CA9-ko cells confirm previous reports for the important role of CA9 in promoting colon tumor cell proliferation. We have further confirmed this in another ongoing CA9-ko study involving lung cancer cells (unpublished data). Importantly, in light of recent concerns raised for the use of tetracylines to achieve gene knockdown [28, 29], our CA9-ko cells provide confirmation that off target effects due to mitochondria dysfunction were not responsible for reduced tumor growth with CA9 knockdown in previous studies. By characterizing these CA9-ko cells in terms of both *in vitro* and *in vivo* growth we believe that they will become valuable tools for future pharmacological development, particularly in the ability to determine potential off-target effects with novel inhibitors.

Due to the established pro-survival role of hypoxia in acidic cell culture conditions, we were keen to investigate if a combination of NHE1 and CA9 removal would lead to greater cell death. Somewhat surprisingly, we observed no increase in cell death for NHE1-ko/CA9-kd cells grown in hypoxic and extremely acidic conditions in the presence of oligomycin. This does however align with previous considerations of the difficulty to achieve an adequately low pH_i_ required to kill a tumor cell as compared to putting it into a static state by disruption of pH_i_ regulating proteins [3]. Therefore it appears that treatment development strategies to slow tumor cell growth based on pH disturbance techniques will require combination with known cellular killing agents to improve efficacy. However, we must interpret 2-dimensional culture results with caution and encourage greater *in vivo* assessment of cell survival pathways in the 3-dimensional tumor microenvironment for pH_i_ disrupting anti-cancer strategies.

3-dimensional spheroids revealed noticeable differences in morphology with NHE1-ko lacking the ability to grow in a compact formation compared to WT cells (Figure 3). This could indicate that NHE1 plays an important role in controlling tumor cell-cell contact mechanisms. Both NHE1 and CA9 have indeed been implicated in a number of studies regarding the migratory/invasive capacity of tumor cells [40, 41] suggesting a role in cell adhesion processes. Unfortunately, colon cancer cell models are essentially non-migratory and are therefore not amenable to adequately test migration. However, the role of NHE1 in cell-cell contact and the ability to grow in 3-dimensions represents an interesting initiation point for future research.

Previously, an analysis of pH_i_ regulation for 8 cancer cell lines revealed a dominant NHE activity in response to acid loading that was reduced upon hypoxia incubation [16]. Hypoxia inhibition of NHE was reversible and did not appear to involve post-translational modification of NHE1 leading to speculation that de-phosphorylation events are linked to a hypoxic inhibition of NHE. We have also observed an apparent hypoxia limitation of NHE1 activity in a survival assays based on resistance to NH_4_Cl pre-pulse acid-loading (Unpublished data). Modification of NHE1 activity by hypoxia would have potential implications for understanding its importance in the tumor microenvironment. However, as strong acute acid-load events such as those induced by NH_4_Cl pre-pulse are unlikely to be experienced within tumors, it appears that NHE1 activity (to maintain resting pH_i_) is indeed fundamental for growth in hypoxia as we observed significant decreases with NHE1-ko in both *in vitro* hypoxia assays and in tumor xenograft experiments. Furthermore, concerns have been raised previously about the acidic pH of the tumor microenvironment limiting NHE1 activity. *In vitro* data suggests acidic pH_e_ levels found in tumors would not substantially limit NHE1 activity [16, 30]. Our tumor xenograft growth studies showing the importance of NHE1 further strengthen the validity for a functional NHE1 within the *in vivo* hypoxic and acidic tumor microenvironment. Comparatively, NHE1 knockout was performed recently in the triple-negative breast cancer model cell line MDA-MB-231 where a strong reduction in tumor xenograft growth was also observed [42]. However, in these breast cancer cells, NHE1-ko strongly reduced tumor formation whereas this was not observed in our colon cancer cell studies indicating that cell specific responses to NHE1 disruptions likely exist. NHE1-ko in breast cancer cell growth has also been recently extended to studies on MCF7 and MDA-MB-231 spheroids [15].

Our use of the aggressively growing LS174 tumor cell xenograft model revealed striking tumor growth reductions in both NHE1-ko and CA9-ko conditions. This ability to use genomic knockout models further reveals the importance of pH regulating proteins in tumor development. Interestingly, the NHE1/CA9-dko tumors grew better than single knockout cells. Protein analysis from tumor extracts revealed a strong induction of another important extracellular CA in cancer, CA12, in NHE1/CA9-dko tumors (Figure 6B). Previously, we have observed increases in CA12 expression in response to shRNA reduction of CA9 expression or CA9 inhibitors [20, 38, 43]. CA12 has also been described to compensate physiologically for CA9 loss in terms of pH regulation and growth [20]. We postulate that the strong induction of CA12 in our NHE1/CA9-dko tumor samples could explain the improved growth rate compared to CA9-ko and NHE1-ko cells. Although, the underlying mechanisms for CA12 induction remain unknown, this is a key event to further explore in the complex network of *intra* and *extra*-cellular CAs and their regulation by pH and/or pCO_2_. This topic is currently under investigation in our laboratory with a series of CA knockout combinations. The fact that we did not observe a strong induction of CA12 *in vitro* with NHE1/CA9-dko (Figure 4) suggests that there are components of the tumor microenvironment, perhaps extracellular acidosis, that play an important role in signaling the induction of compensatory pH regulating proteins. Furthermore, we observed that CA2 was increased in CA9-ko and NHE1/CA9-dko tumor extracts (Figure 6B). This could be explained by the studies from Boron's group describing how directional movement of CO_2_ can be enhanced via a ‘push’ (intracellular CA) or ‘pull’ (extracellular CA) mechanism [44–46]. The capacity for colon tumor cells to induce compensatory CA isoforms in the face of CA9 and/or NHE1 disruption will require consideration in future inhibitor development.

In summary, we have provided cellular knockout models to demonstrate that both NHE1 and CA9 act as important proteins for colon tumor cell progression. This occurs both *in vitro* and *in vivo* however CA9 plays a dominant role in hypoxia while the contribution of NHE1 appears to act independently of O_2_ status. Interestingly, synergistic effects of NHE1 and CA9 combined knockout were not observed *in vivo*, potentially due to a notable increase in CA12 expression which demands further attention in future studies. Our work supports the development of therapeutics targeting pH-regulating proteins as a useful means to slow tumor progression. However, apparent redundancy and induction of compensatory pH regulating proteins must be assessed and the continued generation of genome-altered cell lines for pH-related proteins should greatly benefit drug development.

## MATERIALS AND METHODS

### Cell Culture and reagents

Human colon adenocarcinoma cells (LS174) were originally kindly provided by Dr. Van de Wetering (Utrecht, the Netherlands) and have been verified by DNA profiling. Inducible silencing cells using tetracycline (LS174pTerCA9) were generated and described previously [20]. Cells were maintained under classical culture conditions using DMEM supplemented with 7.5% FBS, penicillin (10 U/mL), and streptomycin (10 μg/mL) and were checked regularly for mycoplasma. Normoxia (Nx) cell culture was performed in a classical 37°C, 5%CO_2_ incubator with hypoxia (Hx) exposure was performed in a Ruskinn workstation with oxygen being replaced by nitrogen to achieve 1% O_2_ conditions.

### Genomic disruption of NHE1 using ZFN

LS174WT and LS174pTerCA9 cells were transfected with Zinc Finger Nuclease (ZFN) plasmids targeting exon 2 of the SLC9A1 (NHE1) gene according to manufacturer protocol (Sigma-Aldrich, Saint-Louis, MO). To increase efficiency of obtaining NHE1 knockout (NHE1-ko) cells, co-transfection of GFP was performed and cell sorting was performed on a FACS ARIA. Sorted cells were allowed 7 days to recover and then the LiCl H^+^-suicide technique was used as described previously [7] to select for NHE1 mutated cells. Individual clones were selected and re-exposed to H^+^-suicide (2x). The NHE1 specific inhibitor HOE694 (100μM) was used as a control in H^+^-suicide manipulations. Other experiments monitoring NHE1 functional activity involved the NH_4_Cl pre-pulse acid-loading and recovery technique described previously [47]. Sequencing of genomic DNA to confirm complete allelic mutations leading to NHE1-ko (Supplementary Figure) was performed using primers provided by Sigma (Primers 5′-3′; F: CAGTCCGACGTCTTCTTCCT; R: TGGATTTGGGTTTGTACCGT).

### Genomic disruption of CA9 using CRISPR-Cas9

LS174WT cells were transfected with PX458 plasmids containing CRISPR-Cas9 targeting regions of the first exon of the carbonic anhydrase 9 (CA9) gene using JetPRIME (Polyplus). The pSpCas9(BB)-2A-GFP (PX458) plasmid was a gift from Dr. Feng Zhang (Addgene plasmid # 48138) [48]. The sgRNA sequence that we cloned into the vector to target CA9 was: 5′ GGGGAATCCTCCTGCATCCG 3′. As the PX458 plasmid contains GFP, cells were first immediately sorted using flow cytometry to obtain cells containing the CRISPR-Cas9. Following this, cells were grown in hypoxia (1% O_2_), labeled with antibodies targeting CA9 and the negative cells were selected to enrich the population for potential CA9 knockout (CA9-ko) cells. Negative CA9 selection was performed twice and followed by classical clonal selection and screening. Sequencing of genomic DNA to confirm the mutations leading to CA9-ko (Supplementary Figure) was performed using lab-designed primers (5′-3′; F: CAGCTCTCGTTTCCAATGC; R: GGAGCCTCAACAGTAGGTAG). NHE1/CA9-dko cells were then created by adding the ZFN targeting NHE1 to confirmed CA9-ko cells and following the selection procedures described above with confirmation via genomic sequencing.

### Cell proliferation and cell death

Cell proliferation was determined in 6-well plates (starting with 2.5×10^4^ cells/well) grown in Nx or Hx over 5 days. At each time point, cells were trypsinized and counted using a Coulter Z1 (Beckman). The cell proliferation index (defined as fold increase) was determined as a fold increase in relation to the cell number obtained 24h (day “0”) following initial cell seeding. In certain experimental manipulations, cells were exposed to varying acidic growth conditions to assess viability following previously described techniques [30]. Cell death was monitored by propidium-iodide (PI) uptake on an ADAM (NanoEnTek) as per manufacturer protocols. ADAM was also used for spheroid proliferation assays (see below).

### Spheroid assays

Three-dimensional spheroid growth assays were performed using the previously described hanging drop technique [20, 24]. Spheroids were grown in media with a pH of 7.4 or 6.7. To monitor spheroid growth and cell viability, spheroids were dissociated with accutase and measured using the Adam as described above. Individual data points involved pooling 8 spheroids per condition. This was performed in duplicate and the experiment was repeated a minimum of three times.

### Western blotting

Analysis of protein expression via Western Blotting was performed as described previously [30]. The primary antibodies used included NHE1/SLC9A1 (Santa-cruz), CA9 (M75, Bayer), CA12 (Sigma), CA2 (Sigma), and HIF1α [49] with ARD1 [50] and β-Actin (Pierce) being used as internal protein loading controls. To improve NHE1 signal, a cell membrane enrichment was performed with centrifugation. LAT1 (KE026 TransGenic Inc.) was used as a loading control for membrane preparations.

### Confocal SNARF pH_i_ measurements

Cells were grown on glass coverslips in hypoxia and fluorescent pH_i_ measurements were performed using a Carboxy-SNARF1-AM (Excitation: 543 nm; Dual emission: 585 nm/640 nm; 15 min dye loading at a working concentration of 10μM) on a confocal microscope as previously described [24]. Recovery from pH_i_ acidification induced by NH_4_Cl pre-pulse was used to monitor NHE1 activity and resting pH_i_ status using previously described buffers [30].

### Tumor xenograft assays

Tumor xenografts were performed as recently described [51] with each modified LS174 cell line (1 × 10^6^ cells) being re-suspended in 300 μL of serum-free DMEM supplemented with insulin–transferrin–selenium (Life Technologies) and injected subcutaneously into the back of 8-week-old female athymic mice (Janvier). Animal care and housing complied with the EU directive and ethical criteria (2010/63/EU) with each cage containing 5 mice in an enriched environment. Food and water were given *ad libitum*, and the litter was changed on a weekly basis. Approval by the local animal care committee (Veterinary Service and Direction of Sanitary and Social Action of Monaco; Dr. H. Raps, Centre Scientifique de Monaco, Monaco) was obtained for the animal experimentation protocol. Tumor dimensions were measured three times a week using calipers, and the tumor volume was determined by using the formula: (4π/3) × *L*/2 × *W*/2 × *H*/2 (*L*, length; *W*, width; and *H*, height). When the tumor volume reached 1000 mm^3^, mice were euthanized, and the tumors were excised. Protein extraction from tumors followed previously described protocols [51].

### Statistics

Data are represented as the mean ± SD or SE (see figure legends for details). Whenever statistical analyses were performed, there was a minimum of 3 experimental replicates. Student's T-tests were performed with statistical significance set at P < 0.05.

We would like to thank the team of Dr. Sylvie Tambutté (CSM) for the use of the confocal microscope to perform pHi measurements and in particular the assistance of Dr. Alex Venn.

## SUPPLEMENTARY MATERIALS FIGURES AND TABLES


