# Supplementary material for: Genetic disruption of the pHi-regulating proteins Na+/H+ exchanger 1 (SLC9A1) and carbonic anhydrase 9 severely reduces growth of colon cancer cells
Source: Oncotarget. 2016 Dec 30;8(6):10225–37. doi: 10.18632/oncotarget.14379 (PMC5354654; doi:10.18632/oncotarget.14379)
Supplement: Supplementary file 1 [file oncotarget-08-10225-s001.pdf]

Genetic disruption of the pH_i-regulating proteins Na^+/H^+ exchanger 1 (SLC9A1) and carbonic anhydrase 9 severely reduces growth of colon cancer cells

SUPPLEMENTARY FIGURES

Clone	Allele	Mutation Type	Mutation Sequence
NHE1-ko#1	1	Insertion	CCGT
	2	Insertion	ACCTTGCTCCTGCCGAGAT GACAAGGAGATCCTGCCC CGGCACTTCGCCCAATAG CAGCCAGTCCCTTCCCGCT TCAGTGACAACGTCGAGC ACAGCTGCGCAAGGAACG CCCGTCGTGGCCAGCCAC GATAGCCGCGCTGCCTCG TCTTGCAGTTCATTCAGGG CACCGGACAGGTCGGTCT TGACAAAAAGAACCGGGC GCCCCTGCGCTGACCGT
NHE1-ko#2	1&2	Insertion	TGG
CA9-ko	1&2	Insertion	A
NHE1/CA9-dko	1&2 (NHE1)	Deletion	C

Supplementary Figure 1: Genomic sequencing of the knockout mutations created in the NHE1 and CA9 genes by ZFN and CRISPR-cas9 targeting respectfully. Mutations in different alleles are noted in the target ZFN region for NHE1 and the CRISPR-cas9 targeting region for CA9.

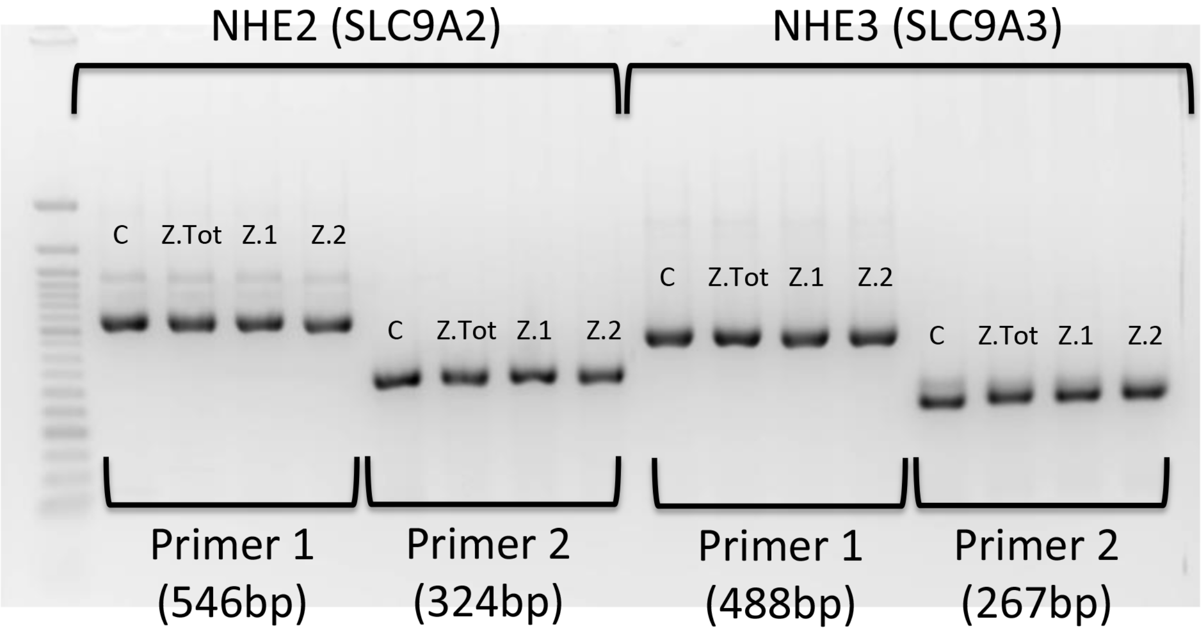

Supplementary Figure 2: mRNA expression of additional NHE isoforms (SLC9A2/3 or NHE2/3) in LS174 WT and NHE1-ko cells. Independent primer sets were used to demonstrate mRNA expression of both NHE2 and NHE3. Abbreviations are given for each band corresponding to the cell type tested. C=LS174 ctl; Z.tot=ZFN NHE1 total population; Z.1=NHE1-ko$^{\#1}$; Z.2=NHE1-ko$^{\#2}$.

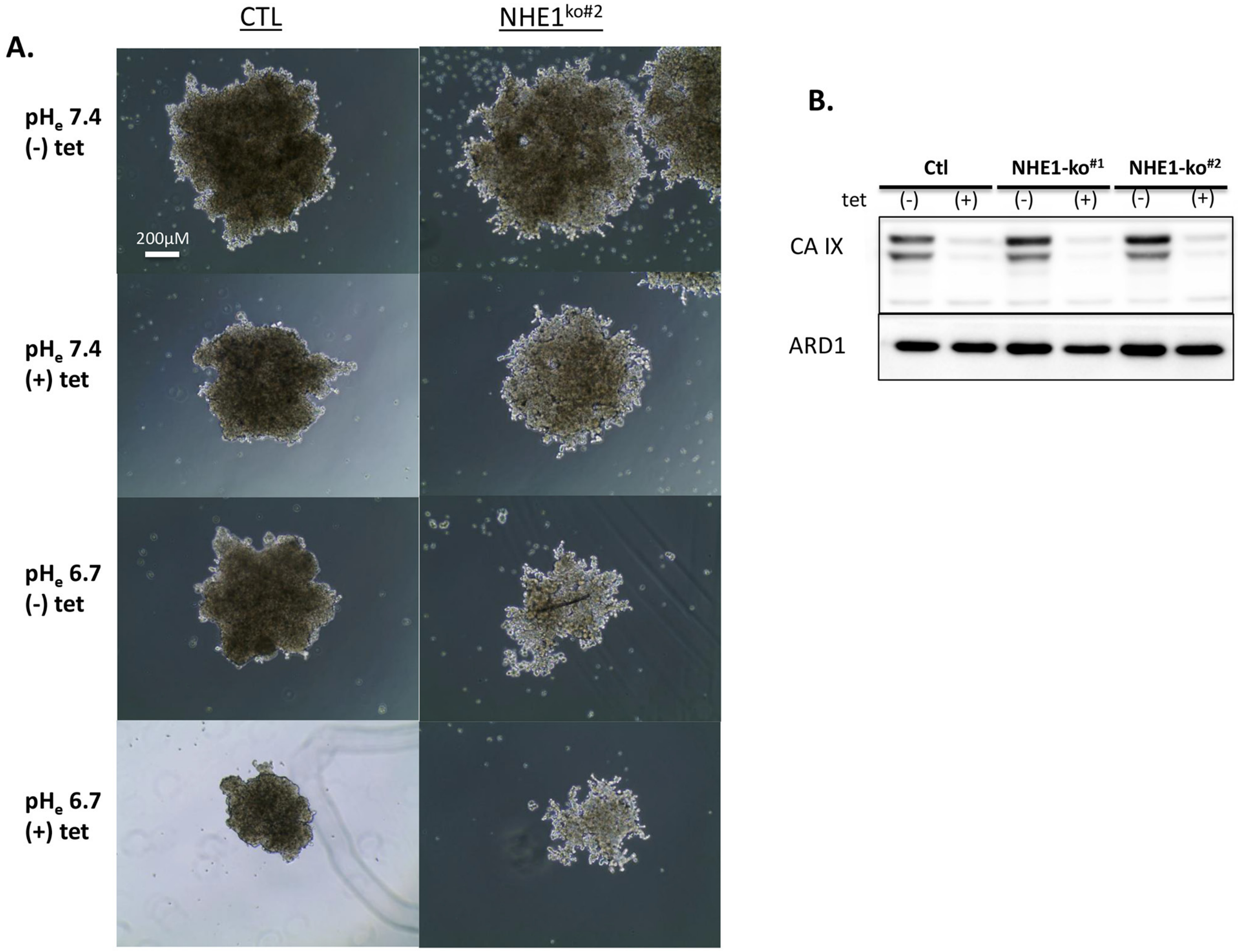

Supplementary Figure 3: 3-D spheroid growth in pH_e of 7.4 and 6.7 demonstrates consistent differences between WT and NHE1-ko cells. **A.** Morphological differences were present with NHE1-ko in both external pH conditions. **B.** WB analysis confirming that CA9-kd is maintained throughout the duration of spheroid growth manipulations.

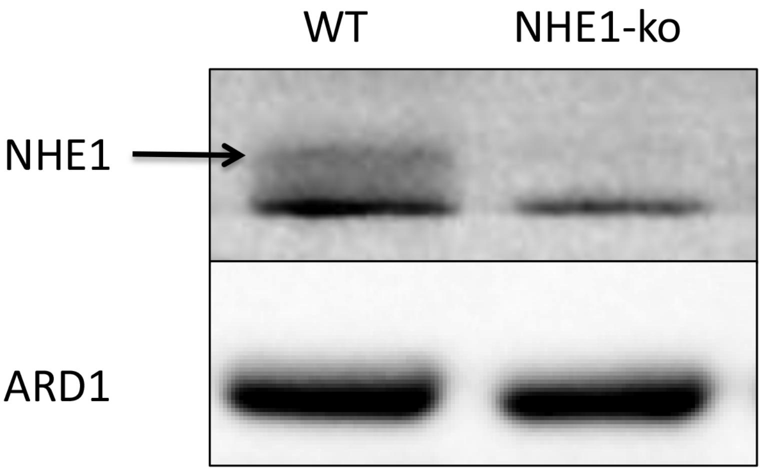

Supplementary Figure 4: WB analysis of tumor extracts from WT and NHE1-ko cells showing maintenance of NHE1-ko in tumor xenograft experiments.
